# The impacts of water quality on the amphibian chytrid fungal pathogen: A systematic review

**DOI:** 10.1111/1758-2229.13274

**Published:** 2024-05-22

**Authors:** Adeline Chew, Matt West, Lee Berger, Laura A. Brannelly

**Affiliations:** ^1^ School of Biosciences The University of Melbourne Parkville Victoria Australia; ^2^ Melbourne Veterinary School The University of Melbourne Werribee Victoria Australia

## Abstract

The pathogenic fungus *Batrachochytrium dendrobatidis* has caused declines of amphibians worldwide. Yet our understanding of how water quality influences fungal pathogenicity is limited. Here, we reviewed experimental studies on the effect of water quality on this pathogen to determine which parameters impacted disease dynamics consistently. The strongest evidence for protective effects is salinity which shows strong antifungal properties in hosts at natural levels. Although many fungicides had detrimental effects on the fungal pathogen in vitro, their impact on the host is variable and they can worsen infection outcomes. However, one fungicide, epoxiconazole, reduced disease effects experimentally and likely in the field. While heavy metals are frequently studied, there is weak evidence that they influence infection outcomes. Nitrogen and phosphorous do not appear to impact pathogen growth or infection in the amphibian host. The effects of other chemicals, like pesticides and disinfectants on infection were mostly unclear with mixed results or lacking an in vivo component. Our study shows that water chemistry does impact disease dynamics, but the effects of specific parameters require more investigation. Improving our understanding of how water chemistry influences disease dynamics will help predict the impact of chytridiomycosis, especially in amphibian populations affected by land use changes.

## Graphical abstract

The effects of water quality and chemicals might impact disease dynamics for frogs. Salinity has a consistent strong impact to reduce disease and nutrients have no impact on disease. There is evidence that the fungicide epoxiconazole reduces disease. There is weak evidence that the heavy metal copper impacts disease. Other water chemicals have unclear or mixed results.

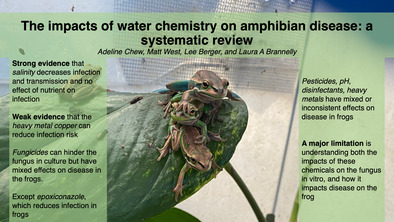

## INTRODUCTION

Amphibians across the globe are threatened by *Batrachochytrium dendrobatidis* (*Bd*), an introduced fungal pathogen that causes the disease chytridiomycosis and has been implicated as a key driver of global biodiversity loss (Scheele, Pasmans, et al., 2019). *Bd* causes structural changes in amphibian skin—disrupting electrolyte balances and leading to cardiac arrest and death in susceptible individuals (Berger et al., 1998; Voyles et al., 2009). Identifying environmental conditions that prevent chytridiomycosis outbreaks is critical for developing effective management actions (Puschendorf et al., 2009; Scheele, Foster, et al., 2019).

Amphibians are often regarded as an indicator of environmental health, showing adverse effects from contaminant exposure (Mann et al., 2009). However, in several cases amphibians were found to inhabit wetlands that contain high levels of pollutants (Sievers et al., 2019; Threlfall et al., 2008). In fact, greater amphibian abundance and richness has occasionally been recorded in some polluted sites compared to more “pristine” areas (Ficken & Byrne, 2013; Lane & Burgin, 2008). These findings indicate that amphibians can persist in contaminated waterways despite the negative effects of anthropogenic pollution on amphibian health. A potential explanation for amphibian occurrence in polluted wetlands is that contaminants are more toxic towards *Bd* (or other parasites) than towards amphibians (Lane & Burgin, 2008). As such, the negative impacts of contamination on amphibian physiology, endocrinology, microbiome, and so forth are sublethal while pathogenic infection might be lethal. Therefore, the suboptimal conditions in polluted water could prevent the persistence of *Bd* or reduce its effects thus providing an environmental refuge for susceptible species by limiting the possibility of disease outbreaks (Lane & Burgin, 2008). However, the effects of water quality and water chemistry on *Bd* and *Bd* infection remain unclear (Haver et al., 2022; Sasso et al., 2021).

Here, we systematically reviewed the literature to examine the effects of water quality on *Bd*: (a) growth and survival in vitro, (b) infection outcomes in amphibian hosts in laboratory experiments, and (c) impact on hosts in the wild. We used the concept of the disease triangle, which describes the relationship between pathogen, host, and environment, to evaluate how water quality affects the spread of *Bd*, particularly in urban settings (Scholthof, 2007). We sought to compile the effects of chemical indicators of water quality (pH, nutrients, and contaminants), provide a summary of *Bd*'s tolerance limits to these parameters, and highlight areas for future research.

## EXPERIMENTAL PROCEDURES

We searched the Web of Science (361), SCOPUS (292), and PubMed (737) databases using the following terms: (pH OR salin* OR conducti* OR contamin* OR extrinsic OR pollut* OR pesticid* OR fungicid* OR insecticid* OR herbicid* OR metal OR nitr* OR phosphor* OR copper OR cadmium OR disinfect* OR magnesium OR zinc OR bismuth OR manganese OR sulph* OR magnesium OR barium OR chlor) *AND* (chytridiomycosis OR batrachoch*). We chose these search terms as they are specific to the chemical parameters that we were interested in while still maintaining scope for a wide range of specific inorganic and organic contaminants to be detected. We restricted our search to publications between 1 January 1998 and 30 December 2023: when chytridiomycosis was identified as a cause of amphibian declines in 1998 (Berger et al., 1998) and when the last search was conducted (last accessed 19 January 2024).

We reviewed articles that described the relationship between at least one indicator of water quality and *Bd* infection. We did not include experiments on *B. salamandrivorans*, another pathogen that causes chytridiomycosis primarily in salamanders (Martel et al., 2013), because most of the impact caused by chytridiomycosis is attributed to *Bd*. We included experimental and observational studies conducted in vitro, in vivo, and in the field and excluded review or meta‐analysis papers. We were interested in how water quality parameters affect either *Bd* physiology (e.g., growth, survival, motility) in culture or *Bd* infection outcomes (e.g., infection prevalence, infection intensity, *Bd*‐induced mortality) in hosts. We screened articles by titles and abstracts and read through the full article if there was any doubt that a paper matched our inclusion criteria based on the title and abstract alone.

We only included experiments on chemicals found in the environment (natural or due to contamination) and excluded experiments on therapeutics. However, we were aware that several experiments on the treatment of chytridiomycosis and decontamination of *Bd* have tested chemical disinfectants and antifungals commonly found in household products that enter the environment through wastewater systems. We included such publications but did not add the terms “disinfectant” or “antifungal” in our search because the terms did not return any relevant publications that were not already included in the search string above.

After identifying all relevant publications for review, we viewed each one in full. We identified the number of publications that have investigated each category of chemical (e.g., herbicide) or parameter (e.g., pH). We also identified the number of separate experiments (i.e., separate studies within a publication) dedicated to each category of chemical or parameter within the experiments. With each publication, we first identified the chemical or parameter of interest and the concentrations used in each experiment. We then identified the control variables relevant to the experimental design: *Bd* strain, *Bd* concentration, chemical application rates, temperature, host species, host life stage, length of experiment, number of sites sampled, sampling period, and location of experiment. Finally, we extracted the key results from each experiment. We summarized the key results by each parameter and each experimental design (i.e., in vitro, in vivo, or field). In vitro effects refer to those on *Bd* in culture. In vivo effects refer to those on *Bd* infection outcomes in hosts under controlled conditions, including laboratory or outdoor mesocosm experiments. Field results included correlations between water quality and *Bd* occurrence in the natural environment and between water quality and *Bd* infection in hosts under natural conditions. For each in vitro, in vivo, and field experiment, we recorded the direction of the effect measured and the concentrations/levels at which the parameter had an effect. If the authors reported the observed environmental concentration of the contaminant(s) of interest in the experiment, we also extracted this data. However, if we were unable to obtain this information from the reviewed articles, we referred to available records in the wider literature for environmental concentrations to assess ecological relevance. We converted all concentrations to μg/L where possible for consistency.

## RESULTS

### Overview

Our search returned a total of 1008 results. From these, we identified 69 publications to include in this review. Most experimental work within the publications were conducted in the United States (52%, *n* = 36) and Australia (25%, *n* = 17). The remaining experimental work took place in Canada, Belgium, Sweden, Brazil, Argentina, Mexico, and South Africa.

**FIGURE 1:**
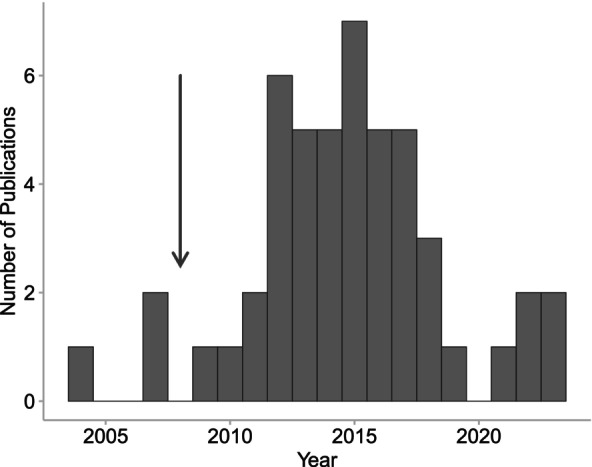
Number of relevant publications by year identified from the literature. The arrow indicates the year, 2008, when it was first suggested that contaminants present in water bodies could provide refugial properties for amphibians against chytridiomycosis.

The earliest publications that fit our inclusion criteria were published in 2003, whereas the greatest number of relevant publications were published in 2012 (Figure 1). Early research was primarily focused on assessing antifungal chemicals against *Bd* and how water quality exacerbates pathogenic infection in hosts (Davidson et al., 2007; Johnson et al., 2003; Johnson & Speare, 2005; Parris & Baud, 2004; Peterson et al., 2007; Piotrowski et al., 2004; Webb et al., 2007). In 2008, the first publication to explicitly test the hypothesis that water quality could provide refuge from *Bd* was published (Threlfall et al., 2008) (Figure 1).

**FIGURE 2:**
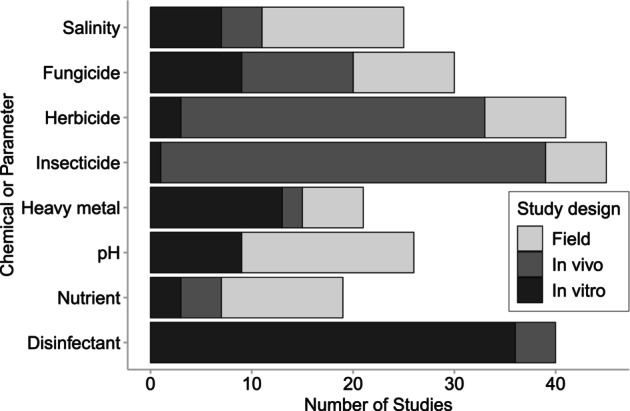
Number of in vitro, in vivo, and field experiments conducted on the effects of each chemical and water quality parameter on *Batrachochytrium dendrobatidis* (*Bd*) and/or *Bd* infection in amphibians. Because some publications used multiple experimental approaches to test the same chemical/parameter or tested the effects of multiple chemicals or parameters, we counted the separate times that each chemical/parameter was tested. In total, there were 248 experiments from 69 publications.

A broad range of water quality parameters have been researched in relation to *Bd* growth and infectivity. Within the 69 publications, 54 individual chemicals or parameters were examined (Table 1). We grouped the parameters identified from our results into nine categories: salinity, fungicide, herbicide, insecticide, heavy metal, pH, nutrient, and disinfectant. Firefighting chemicals were placed within the “disinfectant” category because they were placed within that category in previous publications (Webb et al., 2012). Because some publications used multiple experimental approaches to test the same chemical/parameter or tested the effects of multiple chemicals or parameters, we counted the separate times that each chemical/parameter was tested in a different experimental design as an individual experiment: across the 69 publications, a total of 248 experiments were identified (Figure 2). Salinity has been relatively well studied with effects on *Bd* investigated in the laboratory and in the field in 19 publications containing 26 experiments. Across the 69 publications there has been extensive work investigating the effects of pesticides (fungicides, herbicides, and insecticides) on *Bd* (*n* = 116 experiments); the effects of a total of seven fungicides, four herbicides, and seven insecticides have been described (Table 1). Seventeen heavy metals were explored in 21 experiments and 19 experiments on nutrients (*n* = 13 experiments on nitrogen; *n* = 6 on phosphorus). Experiments that have considered the effects of pH (*n* = 26 experiments) have mostly been field‐based and thus primarily assessed as correlative and observational. There were seven disinfectants including benzalkonium chloride (active ingredient of domestic and industrial cleaning agents), sodium hypochlorite (active ingredient of bleach), and triclosan (antimicrobial agent in personal care and domestic products). These disinfectants were primarily tested in vitro (*n* = 41 experiments, five tested in vivo) and no field‐based experiments have been undertaken.

**TABLE 1:** The specific chemicals or parameters tested and whether their environmental relevance was explained. The chemicals or parameters have been separated into nine broader groups of parameters: Salinity, fungicide, herbicide, insecticide, nitrogen, phosphorous, pH, heavy metals, and disinfectants. We included firefighting chemicals under disinfectants because it does not fit into other contaminant categories and other publications had combined these chemicals. This table represents information found within the publications. The source of the chemical or parameter and the relevant environmental levels are derived from the publications (either measured within the publication or internally cited). Sometimes there was conflicting environmental levels between publications, in which case we reported the highest value here. If no value was provided within the publication for a relevant environmental level, we left the column blank.

	Chemical	Source	Relevant level tested	Environmental level	Number of publications	Citations
***Salinity***	Conductivity	Natural	Yes	2000 μS/cm	8	(Agostini & Burrowes, 2015; Battaglin et al., 2016; Bolom‐Huet et al., 2023; Chestnut et al., 2014; Reeves et al., 2017; Siddons et al., 2020; das Neves‐da‐Silva et al., 2021; Strauss and Smith, 2013)
	Salinity	Natural	Yes	3 × 10‑ μg/L	5	(Heard et al., 2014, 2015; Stockwell, Clulow, & Mahony, 2015; Stockwell, Storrie, et al., 2015; Turner et al., 2021)
	Sodium chloride	Natural	Yes	2 × 10^5^ μg/L	7	(Berger et al., 2009; Clulow et al., 2018; Johnson et al., 2003; Klop‐Toker et al., 2017; Stockwell et al., 2012; Stockwell, Clulow, & Mahony, 2015; Webb et al., 2024)
***Fungicide***	Azoxystrobin	Agriculture	Yes	2 μg/L	1	(Rohr et al., 2017)
	Chlorothalonil	Agriculture	Yes	164 μg/L	2	(McMahon et al., 2013; Rohr et al., 2017)
	Epoxiconazole	Agriculture	Yes	3.4 μg/L	1	(Barbi et al., 2023)
	Mancozeb	Agriculture	Yes	58 μg/L	1	(Rohr et al., 2017)
	Propiconazole	Agriculture	Yes	3.3 μg/L	1	(Barbi et al., 2023)
	Tebuconazole	Agriculture	Yes	0.04 μg/L	1	(Barbi et al., 2023)
	Thiophanate‐methyl	Agriculture	Yes	1720 μg/L	3	(Hanlon & Parris, 2012; Hanlon et al., 2012, 2015)
***Herbicide***	Atrazine	Agriculture	Yes	100 μg/L	6	(Buck et al., 2016; Jones et al., 2017; McMahon et al., 2013; Paetow et al., 2012, 2023; Rohr et al., 2013)
	Glyphosate	Agriculture	Yes	5200 μg/L	9	(Agostini & Burrowes, 2015; Buck et al., 2016; Edge et al., 2011, 2013; Gahl & Houlahan, 2011; Hanlon & Parris, 2012; Jones et al., 2017; Paetow et al., 2012; Romansic et al., 2017)
	Acetochlor	Agriculture	Yes	25.1 μg/L	2	(Buck et al., 2016; Jones et al., 2017)
	2,4‐D	Agriculture	Yes	692 μg/L	2	(Buck et al., 2016; Jones et al., 2017)
***Insecticide***	Carbaryl	Agriculture, residential	Yes	4800 μg/L	8	(Buck et al., 2012, Buck et al., 2016; Cusaac et al., 2021; Davidson et al., 2007; Gaietto et al., 2014; Hanlon & Parris, 2012; Jones et al., 2017; Paetow et al., 2023; Wise et al., 2014)
	Chlorpyrifos	Agriculture	Yes	2.8 µg/L	4	(Agostini & Burrowes, 2015; Buck et al., 2016; Jones et al., 2017; Kleinhenz et al., 2012)
	Cypermethrin	Agriculture			1	(Agostini & Burrowes, 2015)
	Diazinon	Agriculture			1	(Kleinhenz et al., 2012)
	Endosulfan	Agriculture	Yes	9	4	(Agostini & Burrowes, 2015; Buck et al., 2016; Kleinhenz et al., 2012)
	Malathion	Agriculture	Yes	1600 μg/L	5	(Kleinhenz et al., 2012; Reeves et al., 2016; Rumschlag et al., 2014; Rumschlag and Boone, 2015; Wise et al., 2014)
	Permethrin	Agriculture	Yes	17.5 μg/L	2	(Buck et al., 2016; Jones et al., 2017)
***Heavy metal***	Aluminium	Industrial			1	(Love et al., 2016)
	Arsenic	*Not stated*			1	(Peterson et al., 2007)
	Cadmium	*Not stated*			2	(Peterson et al., 2007; Webb et al., 2024)
	Chromium	Industrial			2	(Deknock et al., 2022; Love et al., 2016)
	Copper	Disinfection, fungicide	Yes	7.47 μg/L	7	(Boisvert & Davidson, 2011; Deknock et al., 2022; Gaietto et al., 2014; Parris & Baud, 2004; Peterson et al., 2007; Threlfall et al., 2008; Van Rooij et al., 2017)
	Iron	Industrial			2	(Love et al., 2016; Peterson et al., 2007)
	Lead	Industrial			2	(Love et al., 2016; Peterson et al., 2007)
	Magnesium	*Not stated*			1	(Boisvert & Davidson, 2011)
	Manganese	*Not stated*			1	(Peterson et al., 2007)
	Mercury	Industrial			2	(Love et al., 2016; Peterson et al., 2007)
	Molybdenum	*Not stated*			1	(Peterson et al., 2007)
***Heavy metal***	Nickel	Industrial			3	(Deknock et al., 2022; Love et al., 2016; Peterson et al., 2007)
	Selenium	*Not stated*			1	(Peterson et al., 2007)
	Strontium	*Not stated*			1	(Peterson et al., 2007)
	Uranium	Industrial			1	(Love et al., 2016)
	Vanadium	*not stated*			1	(Peterson et al., 2007)
	Zinc	Industrial	Yes	333.09 μg/L	4	(Deknock et al., 2022; Love et al., 2016; Peterson et al., 2007; Threlfall et al., 2008)
***pH***	pH	Natural	Yes	6.5–9	17	(Agostini & Burrowes, 2015; Battaglin et al., 2016; Bolom‐Huet et al., 2023; Chestnut et al., 2014; Gaertner et al., 2012; Johnson et al., 2003; Kärvemo et al., 2018, 2019; Klop‐Toker et al., 2017; Pauza et al., 2010; Piotrowski et al., 2004; Reeves et al., 2016; Siddons et al., 2020; Stockwell, Clulow, & Mahony, 2015; Strauss and Smith, 2013; Turner et al., 2021; Valencia‐Aguilar et al., 2016)
***Nutrient***	Ammonium	Natural	Yes	100 μg/L	1	(Talbott et al., 2018)
	Ammonium chloride	Fertilizer			1	(Boisvert & Davidson, 2011)
	Ammonium nitrate	Fertilizer	Yes	6430 μg/L	3	(Boisvert & Davidson, 2011; Buck et al., 2016; Dvorsky et al., 2022)
	Ammonium sulphate	Fertilizer			1	(Boisvert & Davidson, 2011)
	Nitrate/nitrite	Natural			4	(Battaglin et al., 2016; Gaertner et al., 2012; Reeves et al., 2016; Strauss and Smith, 2013)
	Phosphorus	Natural			4	(Battaglin et al., 2016; Gaertner et al., 2012; Reeves et al., 2016; Strauss and Smith, 2013)
	Phosphoric acid	Fertilizer	Yes	348 μg/L	1	(Buck et al., 2016)
	Sodium phosphate	Fertilizer	Yes	50 μg/L	1	(Dvorsky et al., 2022)
***Disinfectant***	Benzalkonium chloride	Disinfection			6	(Berger et al., 2009; De Jong et al., 2018; Johnson et al., 2003; Lammens et al., 2021; Webb et al., 2007, 2012)
	Forexpan S	Firefighting			1	(Webb et al., 2012)
	Mixture: Formaldehyde, malachite green, methylene blue (Morenicol FMC‐50)	Disinfection			1	(Lammens et al., 2021)
	Mixture: Peracetic acid, hydrogen peroxide, acetic acid (Wofasteril40)	Disinfection			1	(Lammens et al., 2021)
	Povidone‐iodine	Disinfection			1	(Lammens et al., 2021)
	Sodium hypochlorite	Disinfection			3	(Becker & Gratwicke, 2017; Gold et al., 2013; Johnson et al., 2003)
	Triclosan	Wastewater	No	0.14 μg/L	1	(Brown et al., 2013)

**FIGURE 3:**
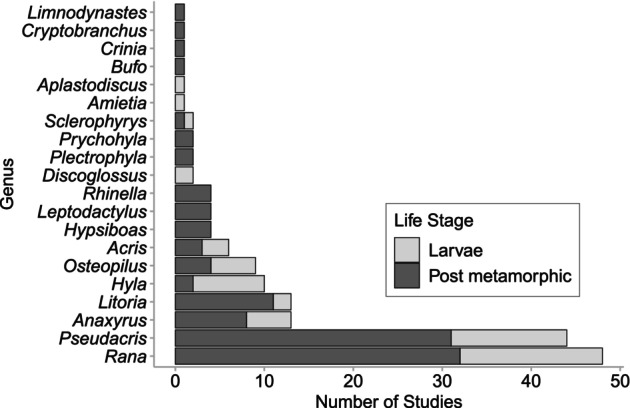
Number of in vivo and field experiments conducted on different amphibian genera across different amphibian life stages. Then, 49 individual published papers conducted experiments on live amphibians from 20 different genera. Some publications used multiple experimental approaches to test water quality parameters on several different species; each experiment listed here is a unique experimental approach, water quality parameter and genera of amphibian for a total of 170 experiments on live animals. Note here that the number of experiments listed is higher than reported for the same experiments in Figure 2, because some experiments explored the same effects on several amphibian species.

Within the in vivo and field experiments, 20 genera of amphibians were explored. The two most common genera involved in these experiments were *Pseudacris* and *Rana* (Figure 3). Most experiments explored the post‐metamorphic adult life stage (66% of experiments).

### Reported chemical concentrations in the environment

Of the 54 chemicals/parameters identified in our review, 21 chemicals/parameters have been studied across at least two study designs (in vitro, in vivo or field) (Table 2). Few publications reported the observed levels of the parameters and contaminants in the natural environment (*n* = 27 chemicals/parameters; Table 1). Some experiments reported different values for the same contaminant in which case we documented the highest concentration. When the environmental concentrations were provided, the concentrations tested within the experiment were typically within the range of observed environmental concentrations (26/27 chemicals; Table 1).

**TABLE 2:** Key results extracted from in vitro, in vivo, and field experiments investigating the effects of different contaminants and water quality parameters on *Batrachochytrium dendrobatidis* (*Bd*) and/or *Bd* infection in amphibians. Only chemicals/parameters reported here have multiple experiments conducted across at least two experimental designs, and the effects have been tested along in the laboratory work. Environmental concentrations of each chemical and parameter are also presented, either presented in the publication itself or found from the wider literature. In vitro key results refer to effects on *Bd* and in vivo key results refer to effects on infection outcomes in hosts. Field key results refer to correlations between the parameter and *Bd* or *Bd* infection in the natural environment. Concentrations at which effects were measured are reported in parentheses. All units are in μg/L except for pH. MIC (minimum inhibitory concentration) is the lowest concentration tested that inhibited *Bd* growth in culture. MLC (minimum lethal concentration) is the lowest concentration tested that killed *Bd* in culture. Blank cells indicated by “–” are areas that have not been studied. “SVL” is snout‐vent length.

	Chemical or parameter	Environmental conc. (μg/L)	In vitro experimental conc. (μg/L)	In vitro key results	In vivo experimental conc. (μg/L)	In vivo key results	Field key results	Citation
***Salinity***	Salinity	Up to 1.30 × 10^7^ (Hopkins & Brodie, 2015)	1560–1.00 × 10^8^	Inhibited growth and killed (MIC = 1.25 × 10^7^ μg/L, MLC = 5.00 × 10^7^ μg/L)	1.00 × 10^6^–5.00 × 10^6^	Decreased infection intensity (1.00 × 10^6^–5.00 × 10^6^ μg/L)	Negatively correlated with infection prevalence and intensity	(Battaglin et al., 2016; Berger et al., 2009; Clulow et al., 2018; Heard et al., 2014, 2015; Johnson et al., 2003; Klop‐Toker et al., 2017; Reeves et al., 2016; Stockwell et al., 2012; Strauss and Smith, 2013; Webb et al., 2024)
***Fungicide***	Azoxystrobin	Up to 2 (Rohr et al., 2017)	0.002–20.6	Inhibited growth (MIC = 0.002 μg/L)	2.06	Increased host mortality and infection intensity (2.06 μg/L)	–	(Rohr et al., 2017)
	Chlorothalonil	Up to 164 (McMahon et al., 2013; Rohr et al., 2017)	0.000176–300	Inhibited growth (MIC = 0.000176 μg/L)	0.0176–30	Increased host mortality (30 μg/L) and infection intensity (0.0176, 17.6, 30 μg/L)	–	(McMahon et al., 2013; Rohr et al., 2017)
	Epoxiconazole	3.4 (Barbi et al., 2023)	1–100	Inhibited growth (MIC = 0.011–0.020 μg/L)	1	Decreased infection intensity when animals were exposed before infection.	Infection prevalence decreased with increasing concentrations in the soil	(Barbi et al., 2023)
	Mancozeb	Up to 58 (Rohr et al., 2017)	0.057–576	Inhibited growth (MIC = 0.57 μg/L)	57.6	Increased host mortality and infection intensity (57.6 μg/L)	–	(Rohr et al., 2017)
	Thiophanate‐methyl	30–1720 (Hanlon et al., 2015)	1500	Inhibited growth (1500 μg/L)	600	Increased larval SVL and mass (600 μg/L)	–	(Hanlon & Parris, 2012; Hanlon et al., 2012, 2015)
***Herbicide***	Atrazine	Up to 250 (Jones et al., 2017)	0.011–212	Inhibited growth (MIC = 0.011 μg/L)	0.18–180	Increased host mortality (65.9 μg/L), decreased infection intensity (1.06, 10.6, 58, and 106 μg/L); no interactive effect on growth/development	–	(McMahon et al., 2013; Paetow et al., 2012, 2023; Rohr et al., 2013)
	Glyphosate	Up to 5200 (Jones et al., 2017)	500	Inhibited growth and survival (500 μg/L)	7–8.64 × 10^4^	Increased host survival (2000 μg/L), decreased infection prevalence and intensity (1000, 2000 μg/L); tadpole mortality (7000 μg/L); no significant effect in other experiments (4200 μg/L)	Negatively correlated with infection prevalence (550, 2890 μg/L), no clear effect (2.16 × 10^4^, 4.32 × 10^4^, 8.64 × 10^4^ μg/L)	(Edge et al., 2011, 2013; Gahl & Houlahan, 2011; Hanlon & Parris, 2012; Hanlon et al., 2012, 2015; Romansic et al., 2017; Cusaac et al., 2021; Paetow et al., 2023)
	Mixture: Acetochlor, atrazine, glyphosate, 2,4‐D	Up to 25.1, 250, 5200, 692, respectively, and individually (Jones et al., 2017)	–	–	4–40 collectively	Decreased infection intensity in *P. regilla* (4 μg/L)	‐	(Buck et al., 2015; Jones et al., 2017)
***Insecticide***	Carbaryl	Up to 4800 (Hanlon & Parris, 2014)	2500	Inhibited growth and survival (2500 μg/L)	10, 300, 480, 500, 3500	Decreased time to tail resorption (300 μg/L)	‐	(Davidson et al., 2007; Buck et al., 2012; Gaietto et al., 2014; Hanlon & Parris, 2014; Rumschlag et al., 2014)
	Malathion	100–1600 (Rumschlag et al., 2014)	–	–	30, 200, 600, 1300	No clear effect	–	(Kleinhenz et al., 2012; Rumschlag et al., 2014; Wise et al., 2014; Rumschlag and Boone, 2015)
***Insecticide***	Mixture: carbaryl, chlorpyrifos, endosulfan, permethrin	Up to 4800, 2.8, 9, 17.5, respectively (Hanlon & Parris, 2012, 2014; Jones et al., 2017)	–	–	4–40 collectively	Increased infection intensity in one species (4 μg/L) and decreased infection intensity in another (4 and 20 μg/L)	–	(Buck et al., 2015; Jones et al., 2017)
	Mixture: chlorpyrifos, diazinon, endosulfan, malathion	Up to 2, 3.30 × 10^4^, 9, 583, respectively (Relyea, 2009)	–	–	4, 75, 0.2, 30 μg/L, respectively	No clear effect	–	(Kleinhenz et al., 2012)
***Heavy metals***	Copper	<0.05–89 (Threlfall et al., 2008)	0.25–1.00 × 10^7^	Inhibited growth (1.00 × 10^5^μg/L of copper sulphate)	0.38–3.18, 12.5	Decreased larval period (0.64–3.18 μg/L), No clear effect at higher concentrations	Sites with higher copper concentrations (1.01–7.47 μg/L) were more likely to be sites with uninfected amphibians	(Boisvert & Davidson, 2011; Deknock et al., 2022; Gaietto et al., 2014; Parris & Baud, 2004; Threlfall et al., 2008; Van Rooij et al., 2017)
	Zinc	10–1063 (Threlfall et al., 2008)	236–4966	No clear effect	–	–	No clear effect	(Deknock et al., 2022; Love et al., 2016; Peterson et al., 2007; Threlfall et al., 2008)
***pH***	pH	6.0–10.2 (Threlfall et al., 2008)	3–10	Inhibited growth (pH 3, 4, 5, 8, 9, 10)	–	–	Inconsistent findings: No significant effect; negatively correlated with infection prevalence and intensity (USA), positively correlated with infection prevalence (Sweden)	(Agostini & Burrowes, 2015; Battaglin et al., 2016; Bolom‐Huet et al., 2023; Chestnut et al., 2014; Gaertner et al., 2012; Johnson et al., 2003; Kärvemo et al., 2018, 2019; Klop‐Toker et al., 2017; Pauza et al., 2010; Piotrowski et al., 2004; Reeves et al., 2016; Siddons et al., 2020; Stockwell, Clulow, & Mahony, 2015; Strauss and Smith, 2013; Turner et al., 2021; Valencia‐Aguilar et al., 2016)
***Nutrient***	Ammonium nitrate	100–6430 (Dvorsky et al., 2022)	0–2.5 × 10^4^	No clear effect	0.437–1800	Nutrients increased amphibian growth but there was no effect on infection	Increased chance of *Bd* detection when there was higher ammonium; Other experiments found no significant effect	(Boisvert & Davidson, 2011; Buck et al., 2016; Dvorsky et al., 2022; Talbott et al., 2018)
	Phosphorus	348 (Buck et al., 2016)	–	–	90–200	Nutrients increased amphibian growth but there was no effect on infection	Increased infection prevalence and intensity with increased phosphorus; Other experiments found no significant effect	(Battaglin et al., 2016; Buck et al., 2016; Gaertner et al., 2012; Reeves et al., 2017; Strauss and Smith, 2013)
***Disinfectant***	F10 Veterinary disinfectant (contains benzalkonium chloride and polyhexalide)	Up to 1900 benzalkonium chloride (Barber & Hartmann, 2022)	1800–1.00 × 10^6^	( Killed (MLC = 15.1 × 10^4^ μg/L); loss of mobility at 1.8 × 10^3^ μg/L	1800	Decreased infection prevalence	–	(De Jong et al. 2018; Webb et al., 2007; Lammens et al 2018)
	Benzalkonium choride	Up to 1900 (Barber & Harmann, 2022)	780 ‐ 1.00 x 10^6.	Inhibited growth (<780 ug/L), Killed (MLC = 1.5 x 10^5	1000	Increased time until death	–	(Berger et al., 2009; Johnson et al., 2003; Webb et al., 2012)
	Triclosan	Up to 2.3 (Kolpin et al., 2002)	10, 100	Inhibited growth (MIC = 100 μg/L)	10, 100, 1000	Increased host survival but reduced change in developmental stage (10 μg/L)	–	(Brown et al., 2013)

### Effects of contaminants and water quality parameters on Bd and Bd infection

#### Salinity

Salinity was measured using different methods and collectively refers to sodium chloride levels or other general salinity measures like conductivity or refractometry. In in vitro experiments, sodium chloride negatively affected *Bd* in four of seven experiments. *Bd* growth decreased in 4.00 × 10^6^ μg/L sodium chloride after 11 days and was completely inhibited at 1.25 × 10^7^ μg/L (as tested by minimum inhibitory concentration [MIC]) after 4 days (Berger et al., 2009; Stockwell et al., 2012). With a 5‐min exposure, *Bd* was killed at 5.00 × 10^7^ μg/L (as tested by minimum lethal concentration [MLC]) sodium chloride (Johnson et al., 2003).

Sodium chloride also limited the effects of *Bd* infection in hosts in three of four in vivo experiments. Infection intensity was significantly lower in *Limnodynastes peronii* tadpoles and *Litoria peronii* juveniles exposed to 1.00 × 10^6^–5.00 × 10^6^ μg/L sodium chloride (Stockwell et al., 2012; Stockwell, Storrie, et al., 2015). Mortality in *Bd*‐exposed *Litoria aurea* adults was significantly lower when exposed to 2.00 × 10^6^ and 4.00 × 10^6^ μg/L sodium chloride and infection prevalence decreased with increasing salinity (Clulow et al., 2018; Stockwell, Clulow, & Mahony, 2015).

Of the 14 experiments that explored the impact of salinity on *Bd* in the field, five experiments reported decreasing infection prevalence and/or intensity with increasing salinity (Bolom‐Huet et al., 2023; Heard et al., 2014, 2015; Stockwell, Clulow, & Mahony, 2015; Stockwell, Storrie, et al., 2015). The other field experiments demonstrated no significant impact of salinity on *Bd* disease dynamics (Table 2).

#### Fungicide

Seven fungicides have been studied (*n* = 9 experiments) at differing concentrations, and all inhibited *Bd* growth in vitro. The fungicides tested were azoxystrobin, chlorothalonil, epoxiconazole, mancozeb, propiconazole, tebuconazole, and thiophanate‐methyl (Table 1). Azoxystrobin (as low as 0.002 μg/L), chlorothalonil (as low as 0.0176 μg/L), and mancozeb (as low as 0.57 μg/L) reduced zoospore abundance compared to controls (McMahon et al., 2013; Rohr et al., 2017). Thiophanate‐methyl was only tested at 1500 μg/L and decreased both zoospore and zoosporangia abundance (Hanlon & Parris, 2012). Another publication tested the MIC for three fungicides and found that *Bd* growth was inhibited at 0.011 μg/L of epoxiconazole, 0.39 μg/L of propiconazole, and 0.010 μg/L of tebuconazole. All these concentrations are within the range found in nature (Table 1).

Exposure to fungicides influenced *Bd* infection in amphibians to differing degrees (*n* = 11 experiments). Exposure to azoxystrobin (2.06 μg/L), mancozeb (57.6 μg/L), and chlorothalonil (17.6 μg/L) exacerbated *Bd*‐induced mortality in *Osteopilus septentrionalis* tadpoles and increased infection intensity in adults (McMahon et al., 2013; Rohr et al., 2017). However, chlorothalonil at low doses (0.176 and 1.76 μg/L) were protective for tadpoles and infection load decreased (McMahon et al., 2013). Thiophanate‐methyl and epoxiconazole had a positive effect on tadpoles that were also exposed to *Bd*. When exposed to 600 μg/L thiophanate‐methyl and *Bd*, tadpoles increased in size, compared to tadpoles exposed to *Bd* only. All tadpoles in the *Bd* and thiophanate‐methyl treatment also tested negative, suggesting that thiophanate‐methyl can help clear *Bd* infection (Hanlon et al., 2012). When tadpoles were exposed to 1 μg/L of epoxiconazole prior to *Bd* exposure, infection prevalence and intensity decreased; however, if animals were first exposed to *Bd* there was no impact of the fungicide on infection (Barbi et al., 2023), indicating that the timing of fungicide exposure is important and that fungicides might be protective but not treat infection. In addition, pharmacokinetic experiments showed that bioaccumulation within newts exposed to realistic field levels could result in epoxiconazole concentrations within the skin above the MIC (Barbi et al., 2023).

Most field experiments (*n* = 10 experiments) found no effect of fungicides on infection. Most showed several fungicides co‐occurring in the amphibian habitat which limits the ability to understand which chemicals influence *Bd* disease dynamics. One experiment found that total fungicide concentrations in sediment samples were higher when infection prevalence and intensity was higher in amphibians (Battaglin et al., 2016). In contrast, as concentrations of epoxiconazole in the field increased, infection prevalence decreased, which was consistent with the in vivo experimental results (Barbi et al., 2023).

#### Herbicide

While many experiments have examined herbicides (*n* = 41 experiments), only two herbicides have been individually tested: glyphosate and atrazine. Just three in vitro experiments have been conducted. Notably 500 μg/L of glyphosate and 106 μg/L of atrazine significantly decreased *Bd* zoospore and zoosporangia growth and survival in vitro (Hanlon & Parris, 2012; McMahon et al., 2013), which are within the environmental ranges for both herbicides (Table 1).

Multiple experiments (*n* = 30) have evaluated the in vivo effects of herbicides on infection in amphibians, but most (*n* = 16 experiments) tested herbicide mixtures. Four experiments examined atrazine effects on infection in amphibians and found contrasting results. One experiment found a significant reduction in infection prevalence with as low as 1 μg/L in tadpoles (McMahon et al., 2013), one found that adult frogs exposed to 65.9 μg/L of atrazine had reduced mortality but their infection of *Bd* was unaffected by the herbicide exposure (Rohr et al., 2013), and two found that there was no significant interactive effect of atrazine and infection on juvenile or larval frogs at 180 μg/L or less of atrazine (Paetow et al., 2012, 2023). There were nine experiments that explored the impacts of glyphosate on disease dynamics in the laboratory, and there appeared to be species‐ and life stage‐specific differences in the impacts of this herbicide. For example, *Hyla versicolor* tadpoles exposed to glyphosate at 2000 μg/L had significantly increased survival compared to those only exposed to *Bd* (Hanlon & Parris, 2014). The same concentration had no clear effect on survival in *Bd*‐exposed *Pseudacris regilla* tadpoles but increasing glyphosate concentrations seemed to decrease infection prevalence and intensity in *P. regilla* tadpoles and *Rana clamitans* juveniles (Edge et al., 2011; Romansic et al., 2017). Higher concentrations (4.2 × 10^3^–2.16 × 10^4^ μg/L) had no effect on juvenile *Cryptobranchus alleganiensis* or *Rana clamitans* infection but was toxic and killed *Anaxyrus americanus* tadpoles (7000 μg/L) (Cusaac et al., 2021; Edge et al., 2013; Paetow et al., 2023). When eight different species were exposed to a mixture containing the herbicides acetochlor, atrazine, glyphosate, and 2,4‐D, *P. regilla* was the only species to experience significantly lower infection intensity at 4 μg/L of the mixture, but surprisingly there was no effect at higher concentrations (Buck et al., 2015; Jones et al., 2017).

Field experiments on different herbicide types (*n* = 8 experiments) found conflicting results. One experiment found that *Bd* was less likely to be detected in soil samples from sites where the total herbicide concentration in the soil were greater (Battaglin et al., 2016). Another experiment found that amphibians were more likely to be positive with higher infection loads in cultivated sites that used herbicides (Agostini & Burrowes, 2015). The other six experiments found no effect of herbicides on amphibian disease dynamics.

#### Insecticide

Forty‐six experiments have been conducted exploring the impacts of insecticides on *Bd* and *Bd* infection in amphibians. However, only one in vitro experiment was conducted: it found that zoospore counts are significantly reduced when exposed to 2500 μg/L of carbaryl (Hanlon et al., 2012). Insecticides carbaryl, chlorpyrifos, diazinon, endosulfan, malathion, and permethrin have been studied in vivo (*n* = 38 experiments) but only three experiments demonstrated an impact of insecticide on infection, and those impacts were not consistent across experiments. Exposure to 300 μg/L carbaryl and *Bd* decreased the time to tail resorption in tadpoles but did not directly impact infection dynamics (Rumschlag et al., 2014). And a mixture of carbaryl, chlorpyrifos, endosulfan, and permethrin increased infection intensity in *Anaxyrus boreas* tadpoles at 4 μg/L but decreased infection intensity in *P. regilla* juveniles at 4 and 20 μg/L (Buck et al., 2015).

In field experiments (*n* = 6), *Bd* zoospore levels in water samples were negatively correlated with neonicotinoid insecticide concentrations (Reeves et al., 2017); however, it is unclear if zoospore levels in the water correlate to infection on an amphibian host (Brannelly et al., 2020). When total insecticide concentrations in the soil were higher, infection and prevalence on the amphibians decreased (Battaglin et al., 2016). However, amphibians sampled in areas that were cultivated and used insecticides were more likely to be infected with higher infection loads than areas that did not use insecticides (Agostini & Burrowes, 2015).

#### Heavy metal

A total of four heavy metals (cadmium, copper, magnesium, and zinc) have been experimentally studied in vitro (*n* = 12 experiments). There was some inconsistency in the effect of copper on *Bd* growth, where one experiment indicated that copper decreased zoospore count at 332 μg/L (copper sulphate) but did not decrease overall growth of the fungus (Threlfall et al., 2008). Another experiment showed that copper did not kill *Bd* but decreased growth at 1.00 × 10^5^ μg/L (Boisvert & Davidson, 2011; Van Rooij et al., 2017). There was no effect of zinc, magnesium, or cadmium on the growth of *Bd* in vitro (Boisvert & Davidson, 2011; Threlfall et al., 2008; Webb et al., 2024). Water samples containing bismuth, copper, chloride, thorium, nitrate, antimony, and manganese were associated with facilitated *Bd* growth in vitro under laboratory conditions while sulphate, selenium, barium, and magnesium in environmental water samples were associated with inhibited *Bd* growth in vitro (Boisvert & Davidson, 2011).

Copper has an impact on amphibians in vivo but does not appear to directly affect fungal infections. When tadpoles were exposed to intermediate concentrations of copper (0.64, 2.12, and 3.18 μg/L) and *Bd* infection, the larval period of the amphibians was shorter compared to no/low copper concentrations, but there was no effect on disease dynamics (Parris & Baud, 2004). At a high dose of copper (12.5 μg/L) there was no significant effect of copper on the amphibians or infection (Gaietto et al., 2014).

In the field (*n* = 6 experiments), 17 heavy metals have been measured and compared with infection prevalence in amphibian populations. Sites with higher concentrations of total heavy metals were more likely to have higher infection prevalence (Love et al., 2016; Peterson et al., 2007). One experiment found no effect of nickel, chromium, or zinc on *Bd* detection in amphibians, but found that amphibians were less likely to be infected with *Bd* at sites with high copper concentrations (1.01–7.47 μg/L) (Deknock et al., 2022).

#### pH

Of the 21 experiments that explored the impacts of pH, nine examined in vitro effects on *Bd*, finding that *Bd* grew optimally at pH 6–7 but was inhibited at pH ≤5 and ≥8 (Johnson & Speare, 2005; Piotrowski et al., 2004). No pH experiments were conducted in vivo.

Correlations between pH and *Bd* infection in field experiments (*n* = 16 experiments) were variable. Field experiments with low pH were correlated with high infection prevalence (Battaglin et al., 2016; Valencia‐Aguilar et al., 2016) and intensity (Battaglin et al., 2016) in amphibians in two studies. But in another, pH >6.5 was correlated with higher infection prevalence in adult amphibians (Kärvemo et al., 2018). Other experiments (*n* = 13) showed that pH had no significant correlation with infection prevalence and intensity.

#### Nutrient

One publication (three experiments) explored the effect of nitrogen fertilizer additives (ammonium chloride, ammonium nitrate, and ammonium sulphate) on *Bd* growth in vitro but found no significant effects (Boisvert & Davidson, 2011). In vivo two experiments explored the impacts of nitrogen on *Bd* infection in amphibians and found that nitrogen did not alter infection in these animals, but nitrogen exposure generally was correlated with increased growth and developmental rates (Buck et al., 2016; Dvorsky et al., 2022). The impacts of nitrogen in the field were variable. Of the seven experiments exploring the impact of nitrogen on infection dynamics in the field, two showed a significant effect, where increases in nitrogen (nitrate or ammonia) in the soil was correlated with increased infection detection, prevalence, and intensity (Battaglin et al., 2016; Talbott et al., 2018).

No in vitro experiments were conducted on the impacts of phosphorous and *Bd*, but two experiments explored the impacts in vivo. Similar to the effects of nitrogen in vivo, there was no impact of phosphorous on disease dynamics, but amphibians exposed to phosphorus with and without *Bd* exposure had increased growth and developmental rates (Buck et al., 2016; Dvorsky et al., 2022). Four field experiments were conducted. Only one experiment showed an effect of phosphorous on disease dynamics with a significant increase in both infection prevalence and intensity on amphibians when there was a higher total phosphorous concentration in the water (Battaglin et al., 2016).

#### Disinfectant

Experiments that assessed the impacts of disinfectants on *Bd* were almost entirely conducted in vitro (*n* = 40 experiments) with four experiments conducted in vivo. Fourteen in vitro experiments explored the impact of benzalkonium chloride (or F10 veterinary disinfectant that contains benzalkonium chloride and polyhexanide), and 15 experiments explored sodium hypochlorite. The MIC and the MLC varied across experiments—here, we report the lowest concentrations that were effective.

The lowest MLC recorded for sodium hypochlorite was 6.00 × 10^5^ μg/L for a 5‐min exposure and 3.00 × 10^5^ μg/L for a 15‐min exposure (Becker & Gratwicke, 2017), while the MIC has not been determined.

The MIC following a continuous exposure of several disinfection products (frequently used for maintaining healthy aquaria) are as follows: 1 × 10^5^ μg/L of Sera pond omnisan (mixture of formaldehyde and malachite green), 1.5 × 10^5^ μg/L of Morenicol FMC‐50 (mixture of formaldehyde, malachite green, and methylene blue), 1 × 10^5^ μg/L of Blagdon pond anti‐fungus and bacteria (mixture of methylene blue, malachite green oxalate, and acriflavine hydrochloride) and 1.3 × 10^7^ μg/L of Povidone‐iodine (Lammens et al., 2021). Forexpan S, while not a disinfectant but a firefighting chemical, was tested and has an MLC of 1.00 × 10^4^ μg/L after a 1‐min exposure (Webb et al., 2007).

Benzalkonium chloride was assessed in vitro and in vivo. In vitro benzalkonium chloride inhibited growth at 780 μg/L after 4 days of exposure, but lower concentrations were not tested (Berger et al., 2009). After 5 min of exposure of *Bd* to benzalkonium chloride, the lowest MLC recorded was 1.78 × 10^4^ μg/L (Webb et al., 2007). In vivo, consistent exposure to 1.00 × 10^4^ for 6 days resulted in increased time until death from chytridiomycosis (Berger et al., 2009). In vitro exposure to veterinary disinfectant F10 at concentrations of 1.8 × 10^4^–7.7 × 10^4^ μg/L for a short time (5 ‐ 120 min) reduced zoopsore motility (De Jong et al., 2018). In vivo, hort daily exposures to F10 (10–15 min per day for up to 9 days) of 5.40 × 10^3^–1.80 × 10^5^ μg/L, was effective at reducing infection prevalence and/or intensity of infection in three amphibian species (De Jong et al., 2018), but lower concentrations for longer exposure periods have not been assessed.

Triclosan (5‐chloro‐2‐(2,4‐dichlorophenoxy)phenol) was also studied in vitro (*n* = 1 experiment) and in vivo (*n* = 1 experiment). In vitro, 10 μg/L was enough to decrease growth (MIC) whereas 100 μg/L completely inhibited growth and killed zoospores (MLC). Exposure to 10 μg/L triclosan did not affect *Bd* infection in tadpoles but increased their overall survival (Brown et al., 2013). Tadpoles that were exposed to both *Bd* and triclosan decreased their developmental time compared to those exposed to *Bd* only (Brown et al., 2013).

### Bd isolates and life stage

Thirty‐one *Bd* isolates have been used across the in vitro and in vivo experiments reviewed here. However, only four experiments compared the responses of multiple strains upon exposure to chemicals (Gahl & Houlahan, 2011; Gaietto et al., 2014; Johnson & Speare, 2005; Piotrowski et al., 2004). No experiment found an effect of strain on fungal growth or survival in vitro or infection dynamics within the host.

The methods for testing in vitro growth and survival were inconsistent across experiments, and thus complicate any comparisons that could be made. Experiments were varied in the fungal life‐stage assessed for in vitro tests, although the walled sporangial life stage seems more resistant than the membrane bound zoospores. For example, copper was observed to reduce zoospore counts (using visual counts on a microscope) at relatively low concentrations (334 μg/L) (Threlfall et al., 2008), but a much higher concentration (>100,000 μg/L, three orders of magnitude higher concentration) (Boisvert & Davidson, 2011) was required to reduce zoosporangia growth and survival. Another example is that thiophanate‐methyl inhibited zoospore but not zoosporangia production when *Bd* was grown on media containing the fungicide (Hanlon & Parris, 2012). Growth conditions were different across in vitro experiments, where temperature and media and growth time differed. We do not yet know the impact of these variable growth conditions on the *Bd* response to contaminants and water parameters.

## DISCUSSION

Our systematic review shows that there has been considerable research on the effects of water chemistry on *Bd* and *Bd* infection in amphibians. In vitro and in vivo experiments indicated that while many water quality parameters have inhibitory or lethal effects on *Bd* cultures, exposure to chemicals resulted in variable infection outcomes in the host. Field experiments demonstrated that only a few parameters influence *Bd* occurrence and infection under natural conditions, and the effects are not always consistent under different ecological contexts.

Salinity has the most evidence for inhibiting *Bd* growth and reducing infection prevalence and intensity under both controlled and natural conditions. Fungicides often had strong inhibitory effects on *Bd* in vitro, even at low doses. However, most fungicides were detrimental to the amphibian when experimentally tested on the host in the laboratory or in the field. The exception is the fungicide epoxiconazole, which was linked to lower prevalence in the field. Overall insecticides and herbicides had inhibitory effects on *Bd* and had some evidence of affecting infection outcomes in amphibians. Many heavy metals have been explored in the field and in vitro but it is unclear if there is an impact of heavy metal at all, although there is some weak evidence that copper might influence *Bd* infection. Nitrogen and phosphorus appear to have little direct impact on *Bd* or on infection in amphibian hosts. Although both low and high pH affected pathogen growth and survival in vitro, the correlations between pH and infection in natural environments were inconsistent. Disinfectants have antifungal properties against *Bd* in vitro, for example, benzalkonium chloride inhibits *Bd* in vitro and in vivo at levels reported in wastewater. However, disinfectants were often not tested in vivo or in the field and therefore the ecological relevance of these findings are limited.

### Salinity

The antifungal properties of sodium chloride are well known and effective against *Bd*. Sodium chloride inhibited growth and zoospore motility at concentrations within natural freshwater salinity ranges (approximately 1.30 × 10^7^ μg/L) (Hopkins & Brodie, 2015) in vitro, and the MLC of sodium chloride decreased with increasing exposure time (Berger et al., 2009; Johnson et al., 2003; Stockwell et al., 2012), indicating that under chronic exposure in natural environments a lower concentration might be sufficient at limiting the fungal pathogen. In vivo experiments supported the in vitro experiments, and sodium chloride at concentrations relevant to freshwater environments (1.00 × 10^6^–5.00 × 10^6^ μg/L) decreased infection intensity in tadpoles and juveniles (Clulow et al., 2018; Stockwell et al., 2012; Stockwell, Clulow, & Mahony, 2015). Field experiments also demonstrated that salt could alter the prevalence of chytridiomycosis under natural conditions as increasing salinity was associated with lower pathogen occurrence in amphibian habitats (Heard et al., 2014, 2015; Stockwell, Clulow, & Mahony, 2015).

The extensive research dedicated to the antifungal effects of salinity on *Bd* across in vitro, in vivo, and field experiments provide strong evidence that salt can protect amphibians against *Bd* infection and provide an environmental refuge. Manipulating salinity in amphibian habitats has been recognized as a promising approach to pathogen mitigation for salt‐tolerant amphibian species (Heard et al., 2018; Scheele et al., 2014; Scheele, Foster, et al., 2019; Stockwell, Clulow, & Mahony, 2015). But there are limits to which salt can benefit amphibian populations. Salt tolerance varies between amphibian species (Kearney et al., 2012), and high salinity environments have the potential to negatively impact even saline tolerant amphibian species, such as reducing their ability to successfully reproduce (Stockwell, Storrie, et al., 2015).

### Fungicide, herbicide, and insecticide (collectively pesticides)

Although all fungicides that have been tested in vitro inhibited *Bd* growth, there was dramatic variability in disease dynamics within the host and observed in field experiments. Individually, the fungicides azoxystrobin, chlorothalonil, and mancozeb, and total fungicides detected in the field exacerbated infection in hosts (Battaglin et al., 2016; McMahon et al., 2013; Rohr et al., 2017); while thiophanate‐methyl offered protection from infection in the laboratory (field data is lacking), and epoxiconazole offered protection in both the laboratory and in the field (Barbi et al., 2023; Hanlon et al., 2012). The strongest evidence for a positive effect of fungicides on amphibians is the laboratory and field results for epoxiconazole (Barbi et al., 2023). While these results are promising, more research on different amphibian species and environments is needed before this fungicide could be used as a mitigation tool. Deliberate use of fungicides for pathogen mitigation needs robust risk assessments due to potential toxicity to ecosystems, as well as potentially subtle health impacts on amphibians (Barbi et al., 2023).

Although herbicides and insecticides inhibited *Bd* in cultures, their effects on infection outcomes in hosts were varied and unclear, showing both positive and negative effects of exposure for the same chemical. Notably, these experiments were conducted using various amphibian species at different life stages. Therefore, the variation in infection outcomes upon exposure to herbicides and insecticides could be attributed to species‐ and life stage‐specific responses. This variability was seen in in vivo experiments, where a single herbicide (e.g., atrazine) was associated with infection progression differently among tadpoles and post metamorphic amphibians (McMahon et al., 2013; Paetow et al., 2012, 2023; Rohr et al., 2013). Because of differences in habitat use, immune responses, and behaviour, there is considerable variation in susceptibility among populations, species, and life stages (Brannelly et al., 2016, 2018; Stockwell et al., 2010), which could explain the context specific differences observed here. Furthermore, the antifungal effects seen in vitro might not apply directly in vivo: for example, if chemicals do not penetrate host epidermal cells, or if bioaccumulation occurs (Barbi et al., 2023; Berger et al., 2005; Roberts et al., 2019; Threlfall et al., 2008) and the pharmacokinetics likely vary with both species and age of the amphibian (Berger et al., 2010). These differences could account for inconsistent findings and underlines the importance of accounting for the role of species and life stage in disease dynamics.

Understanding the effects of mixtures of insecticides, herbicides, and fungicides on infection outcomes provides insight into realistic natural conditions (Relyea, 2009). In several field experiments, the total pesticide concentration was an important predictor of infection dynamics, more so than the presence of individual insecticides, herbicides, or fungicides. A high concentration of agricultural products could be a proxy for agricultural activity and human land use. Disease dynamics and animal health are often found to be affected by agricultural proximity (Brannelly et al., 2015; Brearley et al., 2013; Preuss et al., 2020), indicating that proximity to agricultural activity and land use might be the key driver in the different infection dynamics witnessed in these experiments. However, more individual in vitro and in vivo trials are needed on both individual herbicides and insecticides and in combination to better understand and predict the outcomes in nature. Although these initial experiments addressed realistic scenarios, it remains unclear whether the measured effects on *Bd* infection were caused by the specific combination of chemicals or by just one chemical.

### Heavy metal

While there is scientific conjecture that heavy metals could reduce pathogenic infection as amphibians can thrive in polluted sites (Threlfall et al., 2008), there is minimal evidence that environmental concentrations of heavy metals can inhibit *Bd* infection in hosts. For example, copper in vitro is inhibitory at concentrations four orders of magnitude higher than found in nature (Table 1) (Boisvert & Davidson, 2011) but not at concentrations found in nature (Threlfall et al., 2008). An exception is that one experiment reported a lower probability of finding infected amphibians at sites with high copper levels (Deknock et al., 2022). However, a separate field experiment found amphibians to be more likely infected when there was a history of copper contamination at the site (Love et al., 2016), although actual copper concentrations were not reported in the experiment. These results indicate that the effects of copper contamination are not always consistent. Other heavy metals did not affect *Bd* growth in the laboratory at realistic concentrations (Boisvert & Davidson, 2011; Deknock et al., 2022; Threlfall et al., 2008) and there is no evidence that they affect disease dynamics in the field (Deknock et al., 2022; Love et al., 2016; Peterson et al., 2007).

One field experiment found that contaminated sites with higher total heavy metal concentrations had higher infection prevalence than reference wetlands (i.e., wetlands without a history of mining contamination) (Love et al., 2016). Higher infection with higher levels of environmental contamination was mirrored in the results from sites with higher total pesticide concentrations (Battaglin et al., 2016; Rohr et al., 2017). The presence of contaminants like heavy metals and pesticides are associated with higher human disturbance and land use (Bai et al., 2010; Yang et al., 2018). Anthropological stressors can affect immunity and could increase instances of pathogenic infection in wildlife (Bradley & Altizer, 2007; Brannelly et al., 2019). While the impacts of individual heavy metals on *Bd* and the amphibian host are worth investigating in the field, future research should focus on in vitro and in vivo experiments to demonstrate the mechanism of the impacts of heavy metals on *Bd* disease dynamics at concentrations found in the environment.

### pH

The variability in interactions between pH and *Bd* infection in natural environments provides another example where there are context‐specific factors that influence the environment‐pathogen relationship. Although *Bd* was consistently inhibited at pH ≤5 and ≥8 in vitro (Johnson & Speare, 2005; Piotrowski et al., 2004), the correlation between pH and infection prevalence and intensity varied among field experiments, and there have been no in vivo studies investigating the direct effects of pH on *Bd* infection in the host. In the field, acidity and basicity might indirectly influence *Bd* growth and survival and subsequent infection in hosts by altering the bioavailability of other contaminants in the water column. Bioavailability should be considered in understanding the pathogen–host–environment system and could explain the variability in findings from field experiments (Bryan & Langston, 1992). Heavy metals and pesticides were differently associated with both *Bd* and *Bd* infection under natural conditions across experiments which can be attributed to differences in bioavailability. However, bioavailability of contaminants was rarely considered in these experiments (Edge et al., 2011). Further investigations into how chemical contaminants affect *Bd* at different pH levels and the influence of bioavailability on *Bd* in vitro and in vivo are needed and may help explain the conflicting field results of other chemicals.

### Nutrient

While there were not as many experiments exploring the impacts of agricultural nutrients and fertilizer additives on disease dynamics in amphibians the results are consistent across experiments. Nutrients do not appear to impact disease dynamics directly via both in vitro and in vivo experiments. Furthermore, most field‐based experiments did not find an impact of nutrients on infection dynamics in the amphibians. There were two publications that found a positive association of nitrogen and/or phosphorous concentrations with infection prevalence and intensity (Battaglin et al., 2016; Talbott et al., 2018). However, it is possible that increased infection at these nutrient rich sites is due to land use patterns rather than directly due to the nutrients available. High levels of nitrogen and phosphorous are associated with agriculture and human land use (Bouwman et al., 2013), which is a similar pattern to what we have seen above regarding high levels of pesticides and heavy metals in the environment. If the levels of nitrogen and phosphorous seen here are associated with agricultural land use, then the increasing infection seen in amphibians might be due to confounding factors—for example, higher land use, anthropogenetic change and broad agricultural practice might be correlated with increased infection (Brannelly et al., 2015; Preuss et al., 2020).

### Disinfectant

The inhibitory levels of benzalkonium chloride in vitro and in live animal hosts were lower than concentrations that have been detected in the environment (Tables 1 and 2). Benzalkonium chloride is an organic salt and cationic surfactant known as a quaternary ammonium compound. It is a common ingredient in domestic and industrial products such as fabric softeners and disinfectants commonly entering the environment through wastewater (Barber & Hartmann, 2022). Though environmental levels of benzalkonium chloride vary among countries, concentrations of 1900 μg/L were found in wastewater, with higher levels detected in sediment samples (Barber & Hartmann, 2022). The MIC of benzalkonium chloride has not been determined but the lowest level tested was 780 μg/L which was inhibitory (Berger et al., 2009). Benzalkonium chloride had minimal impacts on disease in the frogs (longer survival time but all animals still succumbed to chytridiomycosis) at levels lower than could be found in wastewater (Berger er al., 2009; Barber & Hartmann, 2022), but the effects on transmission of long‐term exposure of low levels are unknown. It is possible that fungal inhibition occurs in some sites contaminated by sewerage or other waste, because higher amphibian abundance and diversity of amphibians have been reported at urban sites (Lane & Burgin, 2008). Veterinary disinfectant F10 (containing benzalkonium chloride and polyhexanide) in short exposure time periods reduces infection in amphibian hosts and motility at concentrations that are higher than found in wastewater (De Jong et al., 2018), but effects of long‐term exposure of low levels are unknown.

Sodium hypochlorite is also a commonly used disinfectant which likely acts by damaging cell membranes, enzymatic activity, and DNA (Cashins et al., 2008; Fukuzaki, 2006). It is used in wastewater treatment plants and is the active ingredient in household bleach. The inorganic compound releases chlorine when in water (Fukuzaki, 2006). Chlorine levels in discharged wastewater can be between 100 and 200 μg/L (Manduzio et al., 2004), which is three orders of magnitude lower than known lethal levels of sodium hypochlorite in vitro, but the MIC has not been determined (Becker & Gratwicke, 2017). As with benzalkonium chloride, it would be useful to test low concentrations with longer exposure periods and understand how chronic exposure can reduce *Bd* growth and fitness.

Because the research on benzalkonium chloride and sodium hypochlorite were aimed at evaluating the effectiveness of the compounds either as treatment options in hosts or as disinfectants for laboratory and field equipment, there is no field data indicating that actual infection in amphibians is impacted by these chemicals at concentrations found in nature. However, because these compounds are common contaminants, we suggest that further research on both lethal and sublethal effects of environmental levels of benzalkonium chloride and sodium hypochlorite on *Bd* is warranted, as well as the effects of chronic exposure to these disinfectants on the amphibian host.

While triclosan inhibited *Bd* in vitro, the disinfectant had contrasting lethal and sublethal effects in hosts. Triclosan is a phenolic compound used in household products for its antimicrobial properties and was found at concentrations up to 2.3 μg/L in streams (Kolpin et al., 2002; Singer et al., 2002). Given that the lethal dose of triclosan after 6 days of exposure was 43 times the maximum environmental concentrations, it is unlikely that the chemical kills *Bd* in the environment. However, concentrations 4.3 times the environmental levels resulted in decreased growth of *Bd* in vitro, and while infection in tadpoles was not affected by that concentration, their survival increased, indicating an increased tolerance of infection when exposed to triclosan. There was a cost of triclosan exposure where tadpoles exposed to *Bd* and triclosan had halted development (Brown et al., 2013). This example highlights the complexities and potential costs of contaminant exposure to the host. Even if there is a benefit of reduced infection or increased survival when the contaminant is present, it is also necessary to recognize the sublethal physiological and morphological effects of contaminants on the host.

### Limitations and further recommendations for standardized approach

Previous in vitro, in vivo, and field experiments allow us to identify important chemicals/parameters and the levels at which they consistently influence *Bd* and infection outcomes in hosts. However, variability in the specific aspects of methodology limits our interpretations of important water quality parameters and their effects.

#### Comparison of multiple Bd isolates

With over 30 *Bd* isolates used across in vitro and in vivo experiments, inconsistent antifungal results could be due to differences in strain phenotype. We know that different strains have different growth patterns and parameters that often reflect their environment. For example, strains from cool adapted regions can grow at cooler temperatures (Stevenson et al., 2013); therefore, it is possible that the local *Bd* might adapt and be less susceptible to that chemical/parameter at local levels. Only four experiments compared the responses of multiple strains upon exposure to chemicals (Gahl & Houlahan, 2011; Gaietto et al., 2014; Johnson & Speare, 2005; Piotrowski et al., 2004), and none showed a difference in response to the chemical parameter.

#### Comparison of effects on different Bd life stages

In vitro experiments rarely addressed the effects of water quality on separate *Bd* life stages. Zoospores are the motile waterborne stage, and their vigour could impact dispersal, transmission, and infectivity (Berger et al., 2005; Voyles et al., 2009). On the other hand, zoosporangia growth and survival influence its reproductive capacity and subsequent release of new zoospores (Voyles et al., 2012). Greater susceptibility of *Bd* zoospores to chemicals might be because they lack a cell wall whereas the walled zoosporangia are more protected (Berger et al., 2005). For example, a low saline environment was shown to reduce *Bd* transmission in tadpoles (likely through inhibition of the infective zoospores) but did not directly impact infection levels within the animal (Clulow et al., 2018). While a saline environment might not “treat” infection, lower transmission rates can lead to increased survival in the host species. It is important to consider how each water quality parameter can differently affect the life stages in in vitro experiments to help clarify the underlying mechanisms that drive *Bd* occurrence in the environment. Even if a chemical does not completely inhibit the growth of the fungal pathogen, a reduction in transmission or growth could affect prevalence and mortality rates.

#### Incorporation of in vitro, in vivo, and field approaches and study design recommendations

Research on both the host and the pathogen in vitro and in vivo is required before we can accurately describe the cumulative effects of water chemistry on *Bd* and infection outcomes in the field and determine if there are chemical refugia for amphibians affected by *Bd*. Of the 54 chemicals tested, less than half (*n* = 21) were assessed across multiple study designs (Table 2). Because salinity has been studied across in vitro, in vivo, and field experiments, we have a clear understanding of its interaction with *Bd* both independent of and in hosts: it reduces infection dynamics/impacts in the host. However, over 26 experiments across 19 individual publications were conducted in order to clarify it's impact on infection in the host. For fertilizer nutrient additives nitrogen and phosphorous, while there are relatively few studies, the evidence is consistent that these additives do not directly impact fungal growth or infection dynamics in the host, although high nutrient sites might affect infection dynamics in the field. However, key gaps in knowledge remain with regard to pesticides, disinfectants, heavy metals, and pH. For example, there is evidence that one fungicide, epoxiconazole, explained lower *Bd* prevalence in the field, with results that were supported in vitro and in vivo (Barbi et al., 2023). However, it was examined in only one publication, and not repeated under different ecological contexts. Other chemicals like disinfectants or heavy metals have not been tested repeatedly or under different ecological circumstances, making broad ecological recommendations difficult. And pH has no in vivo experiments; therefore, it is difficult to understand the discrepancies between the clear results in vitro, where the pathogen dies at high and low pH, and the variable results from the field experiments. Future research on the other water quality parameters should take a similar approach—namely, to fill in the gaps where in vitro, in vivo, or field knowledge are lacking.

Understanding the nuanced impacts of water chemistry on infection and disease dynamics can be complex. And understanding the effects of these chemicals/parameters are difficult when each experiment or publication is conducted independently or answer only one part of the larger picture. Based on our critical assessment and collation of the previous research we have developed key suggestions for designing comprehensive water chemistry studies exploring the impacts on *Bd* and host impacts. (1) Understand the history of contaminant use in nearby industries in terms of the chemicals used, the timing of release, and it's persistence in the environment. (2) Measure the priority chemical levels in the water and in the sediment at time intervals that reflect amphibian life stages and when they would be exposed to the chemical/parameter. (3) In vitro studies must investigate both MLC and MICs at chronic exposures. Both zoospore and zoosporangia life stages should be assessed using standardized methods. (4) In vivo lab studies should explore the effects of realistic concentrations at chronic exposure levels, and include potential for chemical bioaccumulation. Exploring the impacts of pH and chemical exposure is also needed because pH affects bioaccumulation. (5) Field studies should include multiple control sites and correlate the impacts of chemical/parameter levels to multiple parameters of disease dynamics such as infection prevalence, infection loads, and population abundance. *Bd* infection is seasonal, so sampling efforts need to incorporate and account for the seasonality of the disease. Disease dynamics should be sampled from live amphibian hosts, and not rely on eDNA sample collection to detect the pathogen, because it is not a reliable technique (Brannelly et al., 2020). Field studies should also consider covariates that might further explain the relationship between contaminants and infection dynamics, such as land use patterns. For example, agricultural land use is known to be correlated with increased disease; therefore, to understand the impacts of particular agricultural contaminants, parsing the direct impacts of contaminants from the impacts of land use will need to be considered. (6) If possible, field interventions can provide the final evidence for determining the effect of a manipulative single factor, such as the manipulation experiments with salinity (Stockwell et al, 2015).

### Conclusions

We reviewed the published empirical experiments on the effects of water chemistry on *Bd* and chytridiomycosis to better understand the ecology of the pathogen and disease dynamics. The heterogeneous occurrence of *Bd* and *Bd* infection in the environment is difficult to explain but in vitro and in vivo experiments suggest that this can be caused by chemical interactions that can influence the fungal pathogen and its hosts. The variability in impacts of pollutants and various chemicals on amphibian hosts highlights the complexity of understanding the effects of water chemistry on host‐pathogen interactions. While a chemical could reduce *Bd* growth, it might have a more detrimental impact on amphibians making the host more vulnerable to infection. Adverse effects of environmental pollutants such as pesticides and heavy metals on amphibian health have been well documented, with sublethal effects including teratogenesis, immunosuppression, altered behaviour, and reduced size (Egea‐Serrano et al., 2012; Mann et al., 2009). Therefore, it is crucial to consider the impacts of pollutant exposure on both host and pathogen biology to understand the mechanisms by which contaminants and the pathogen synergistically or antagonistically interact.

While there are some clear patterns across experiments with certain water quality parameters (e.g., increased salinity decreases infection, nutrients have little impact on infection outcomes), there are water quality measures that can cause variable outcomes under different ecological contexts. Therefore, repeated experiments conducted through a combination of experimental designs (i.e., in vitro, in vivo, and field experiments) and repeated assessment under different ecological contexts (e.g., different host species, different locations) is crucial for broad characterizations to be drawn. Improving our understanding of the chemicals that influence chytridiomycosis will help identify environmental conditions that can either prevent or exacerbate pathogen impacts for susceptible amphibian species. Ultimately, this knowledge could be used to help derive new strategies for controlling and mitigating this devastating fungal pathogen.

## AUTHOR CONTRIBUTIONS

**Adeline Chew:** Conceptualization (equal); data curation (equal); investigation (equal); visualization (equal); writing – original draft (equal). **Matt West:** Conceptualization (equal); supervision (equal); writing – review and editing (equal). **Lee Berger:** Conceptualization (equal); supervision (equal); writing – review and editing (equal). **Laura A. Brannelly:** Conceptualization (equal); data curation (equal); supervision (equal); validation (equal); visualization (equal); writing – original draft (equal).

## CONFLICT OF INTEREST STATEMENT

The authors declare no conflict of interests.

## Data Availability

No data were directly collected for this systematic review.
